# Methodologic approach to sampling and field-based data collection for a large-scale in-depth interview study: The Social Position and Family Formation (SPAFF) project

**DOI:** 10.1371/journal.pone.0210776

**Published:** 2019-01-15

**Authors:** Diana Romero, Amy Kwan, Lauren Suchman

**Affiliations:** 1 Department of Community Health and Social Sciences, CUNY Graduate School of Public Health and Health Policy, New York, New York, United States of America; 2 Institute for Global Health Sciences, University of California, San Francisco (UCSF), San Francisco, CA, United States of America; University of California Irvine, UNITED STATES

## Abstract

Over the past several decades there have been dramatic shifts in demographic patterns pertaining to family formation, with declining and delayed marriage and childbearing, and increased cohabitation in the United States and other Western industrialized nations. These trends in family demography have been predominantly studied using large-scale datasets, which have identified total population and subgroup trends over time, including differences by age, gender, racial/ethnic, economic, educational, religious, and other characteristics. However, there is limited knowledge and understanding of how individuals across different levels of social position, as well as other important characteristics, make decisions around forming families. This lack of qualitative data on contemporary attitudes regarding family formation has hampered our ability to more completely understand the factors driving behaviors pertaining to the large-scale (ie, international) shifts in demographic trends. The Social Position and Family Formation (SPAFF) project is an in-depth interview study that used quantitative data to guide recruitment of a large sample for qualitative interview data collection on factors influencing different aspects of family formation among heterosexual females and males (18–35 years) in the context of individuals’ social position. This methodological paper describes the use of a ‘quantitatively-informed’ purposive sampling approach in a large metropolitan area to collect qualitative data (through in-depth interviews) from a large sample (n = 200), utilizing web-based tools for successful community-based recruitment and project management.

## Introduction

The field of family demography has been extensively studied using large-scale datasets from both observational surveys [1,2] as well as surveillance or administrative datasets [3]. These research approaches have been able to identify trends over time, including important differences by subgroups (e.g., age, gender, racial/ethnic, economic, educational, religious, etc.). Indeed, from these type of research activities, we know that over the past several decades, many Western industrialized countries have witnessed a dramatic shift in the phenomena associated with family formation, in particular with regard to marriage, cohabitation, and childbearing. In the United States (US), marriage rates have been on a steady decline, divorce has decreased, cohabiting unions have increased markedly, and childbearing overall has decreased [4–11]. Alongside these trends has been increasing attention directed at rates of childbearing outside of marriage, which has risen from 4% of all births in 1940 to about 40% in each year from 2007 to 2013 [12–18]. The implications of these demographic trends are broad-based, however, they are most salient for individuals at the lowest ranks of income distribution. Although strong persistent associations exist between low socioeconomic status and single parenthood [19], these findings from predominantly quantitative research still leave us uncertain of the specific mechanisms that may be at work to explain these associations.

Of particular note is the dearth of information about *how* individuals (poor and non-poor) make decisions around forming families. The lack of studies focusing on attitudes regarding family formation has hampered our ability to more completely understand the factors driving behaviors pertaining to intimate relationships and childbearing. The Social Position and Family Formation (SPAFF) project is a large-scale interview study that has sought to address this deficiency by collecting data via open-ended, in-depth interviews (IDI) with individuals from diverse backgrounds and characteristics in an urban setting. For this study, the sampling approach was informed by New York City (NYC) population-level data in order to recruit individuals from neighborhoods that have similar characteristics to those in the city overall. We explore factors influencing family formation with a particular focus on intimate relationships (e.g. dating, cohabitation, and marriage) and childbearing, in the context of different aspects of individuals’ social position (e.g. income, education).

Since the SPAFF study centers on family formation and social position, the sample needed to reflect individuals across the socioeconomic (SES) spectrum. Thus, our goal was to recruit a large, diverse sample to permit analysis both within and across SES groups while taking into account other characteristics, such as relationship and parenting status, age, and gender. Undertaking a large-scale IDI study in a large urban setting necessitated considerable planning for the field component of the study, including determination of the sampling frame and recruitment locations; training, monitoring and communication with interviewers and other field staff; and transfer and processing of the data. We anticipated that the plethora of internet-based tools in communication and management, in general, might prove useful for our purposes in managing a large-scale field effort. However, there is a dearth of literature focusing on the use of technology in general, and web applications specifically, to facilitate sample recruitment and project management within the context of a large IDI study [20–29].

The aim of this paper is to describe a study that employed a combination of field-based implementation activities to recruit and interview a large sample of young adults on family-formation in an urban setting. As described by Johnson et al [30], research can take on various forms of “mixing” of qualitative and quantitative components (e.g., theoretical perspective, sampling design, data collection and analysis) [30]. The qualitative-dominant paradigm “relies on a qualitative, constructivist-poststructuralist-critical view of the research process, while concurrently recognizing that the addition of quantitative data and approaches are likely to benefit most research projects” [30]. In light of the sparse literature on use of web-based tools for sampling, recruitment and management of large qualitative research projects, we turned to the various applications available on the internet to assist with the implementation of our study. As such, we present a detailed account of the integrated use of multiple web-based tools to recruit a diverse sample from the larger NYC metropolitan area (which includes northern New Jersey [NJ]) and streamline qualitative data collection. We non-randomly selected neighborhoods with demographic characteristics broadly similar to most NYC boroughs, and we purposively sampled respondents of those boroughs with aggregate socioeconomic attributes consistent with residents of each neighborhood. Therefore, our findings are not generalizable to the resident population of each neighborhood, but the results may be less biased than if we purposively sampled respondents without any consideration of neighborhood and city composition. The focus of this methodological paper is on the ‘quantitatively-informed’ purposive sampling approach and the technological elements that were leveraged to efficiently advance qualitative data collection for a project studying social position in the context of family formation. Results of substantive analysis of the data collected are being reported separately.

## Methods

The SPAFF project is a study that set out to conduct IDIs with a diverse sample of 200 women and men between 18 and 35 years of age. This required developing a sampling strategy that would increase the likelihood of recruiting participants in different neighborhoods with a *range* of characteristics and who would, therefore, reflect individuals across the socioeconomic (SES) spectrum and the demographics of the broader NYC metropolitan area. Put differently, we sought to minimize the potential bias associated with the correlation between individuals’ characteristics and the neighborhoods they live in or frequent (e.g., more vs. less affluent neighborhoods, or those with more members of a particular ethnic or religious group). In addition, in order to efficiently carry out such a large qualitative data collection effort, we designed the study to incorporate various web-based tools to assist with the purposive sampling, recruitment, data collection and management, and analysis phases. Below we describe the various elements of the study design and field-based methods.

### Sampling strategy

**Fig 1:**
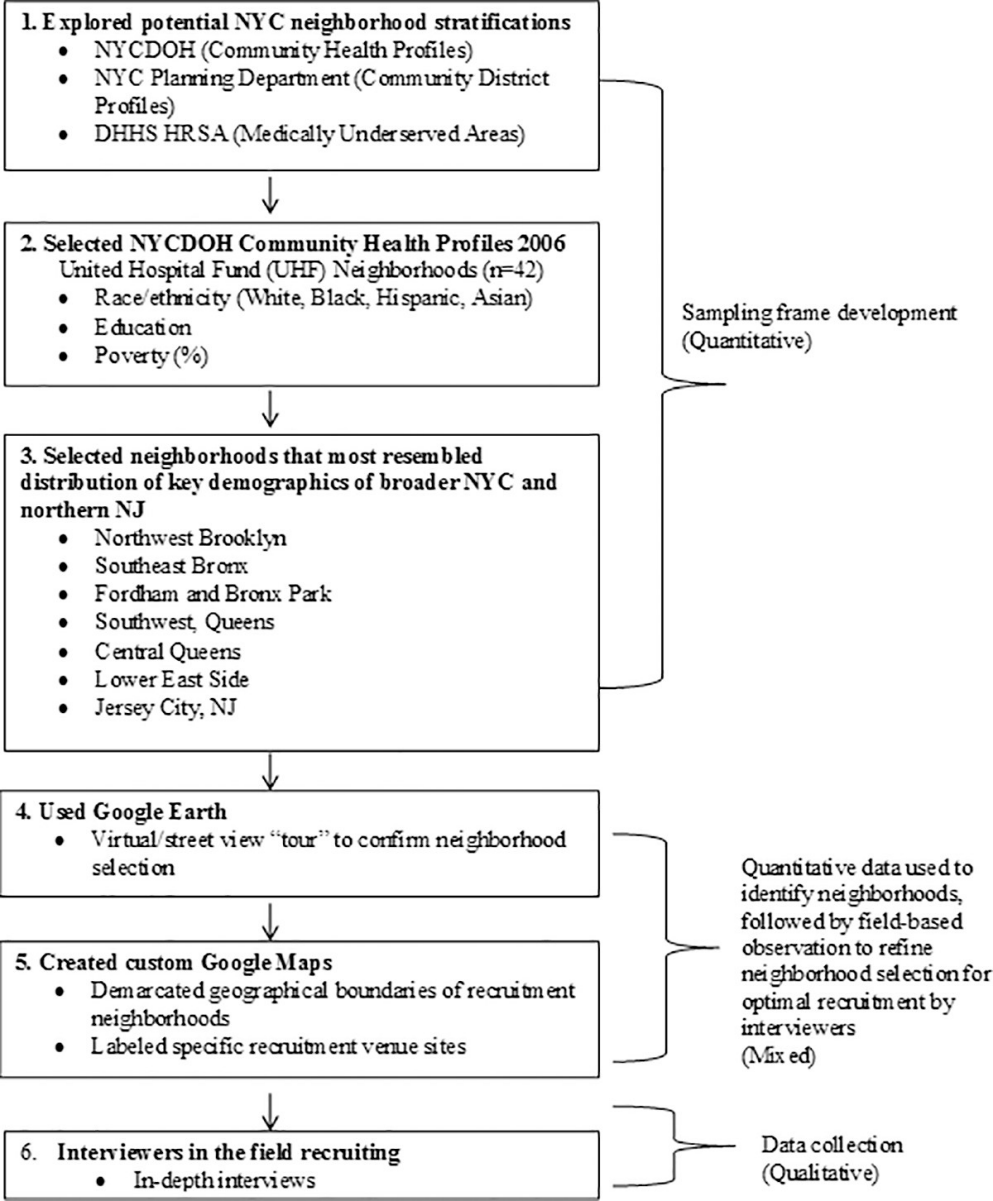
Sampling strategy for neighborhood identification.

We employed a quantitatively-informed approach to determine the sampling frame from which participants would be recruited (Fig 1). First, we identified possible NYC sources of key demographic (i.e. race/ethnicity, foreign born, poverty) data by geographic area. The Community Health Survey (CHS) from the NYC Department of Health and Mental Hygiene was the best source of these data, as they were broken down by 42 NYC neighborhoods (http://www.nyc.gov/html/doh/html/data/data2006.shtml). We examined CHS data on race/ethnicity, percent foreign-born, and percent living below the federal poverty line, across all 42 neighborhoods (data not shown). Neighborhoods that had demographic characteristics similar to that of NYC overall were non-randomly selected. For example, the racial/ethnic distribution in NYC was 35% White, 27% Hispanic, and 24% Black, with 36% foreign-born and 21% below poverty. We compared the distribution in each neighborhood and identified 1–2 neighborhoods within the boroughs of the Bronx, Manhattan, Queens and Brooklyn that closely resembled the NYC distribution in approximately two of these demographic characteristics. The borough of Staten Island (SI) was excluded from the sampling frame due to its distinctly different geographic, sociopolitical, and demographic characteristics (e.g., 71% white; 12% Hispanic; 9% black; 10% below poverty) as compared to NYC overall. Specifically, we could not identify individual neighborhoods in SI that had a similar racial/ethnic and income *distribution* to that of NYC, which would likely generate a diverse sample of individuals.

Similarly, data from the 2006–2008 American Community Survey were used to select the northern NJ site, which is considered part of the larger NYC metropolitan area given the extensive amount of bi-directional commuting between Jersey City and NYC. Moreover, inclusion of a site in NJ would permit sub-group analyses (beyond the scope of this paper) comparing family-formation attitudes among low-income young adults given different state welfare policy regimes in NY and NJ. The final sampling frame included neighborhoods in four boroughs of New York City (the Lower East Side in Manhattan, Northwest Brooklyn, Southwest and Central Queens, Fordham and Bronx Park in the Bronx) and Jersey City, New Jersey.

The non-random selection of boroughs resulted in a purposive sample of respondents similar to the demographic characteristics of each geographic neighborhood. The goal was to recruit 200 participants between the ages of 18 and 35 from these neighborhoods who spoke English or Spanish as their primary language. We sought a roughly equal distribution of men and women across the socioeconomic spectrum (income and education), as well as major racial/ethnic categories (Black, White, Latino) that had been focused on in previous demographic research. In addition, we wanted to ensure that the sample had ample variation across relationship categories (e.g. single, cohabiting, married, etc.) and among respondents who did and did not have children. Thus, this sampling strategy generated a large IDI sample necessary to ensure saturation of themes in the data and allow for sub-group analyses by the various population characteristics of interest. While this provides favorable conditions for credibility (or confidence in the truth) of the findings, [31] that is addressed in the substantive analyses reported separately.

The research team visited each of the field sites and identified potential recruitment venues, such as tax preparation offices, laundromats, hair salons, fitness centers, public libraries, and cafés. We sought to recruit individuals from settings that would permit us to draw a community-based sample without the potential bias associated with recruiting in partnership with specific organizations. A short screener survey was developed for interviewers to determine an individual’s eligibility to participate in the project. The screener collected demographic information relevant to the study such as gender, age, relationship status, household income, and location of residence.

Principles of human subject protection were part of the interviewer training sessions, including comprehensive provision of study information so that potential participants could make informed decisions regarding participation and provide written informed consent if they decided to do so. Given the sampling approach described above, we monitored enrollment by sex, income level, and relationship status in order to obtain a distribution across relevant categories to allow for subgroup analyses. This meant that additional follow-up efforts were carried out to schedule interviews with certain groups such as men, African American/black, older and married individuals, The research protocol was approved by the City University of New York (CUNY) Institutional Review Board (Protocol #337386–2).

### Interview guide

**Fig 2:**
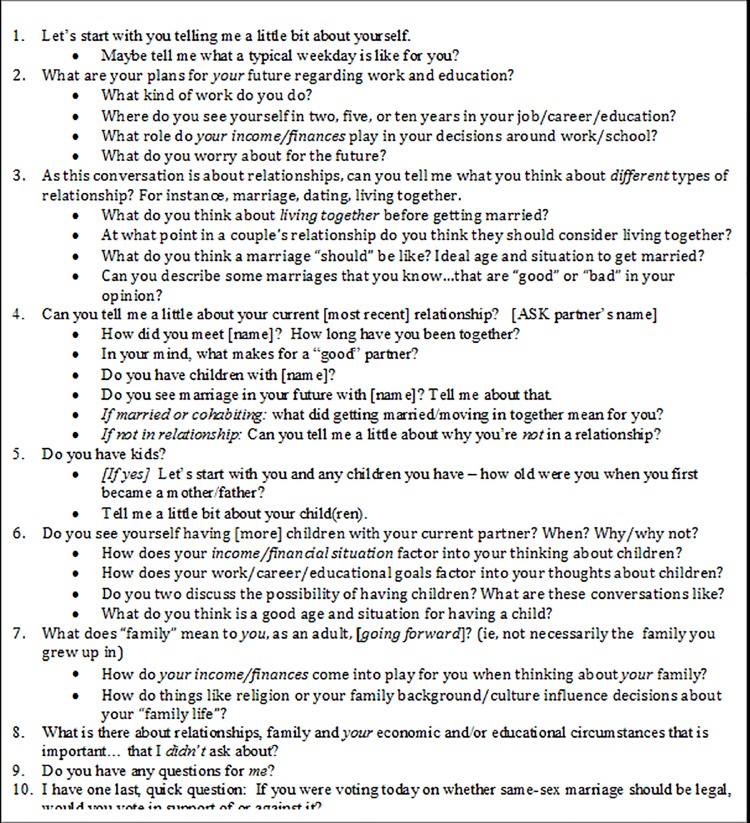
SPAFF study interview guide.

A number of previous studies on topics related to family-formation were consulted and ultimately informed the development of the interview guide [5,21,32–34]. These studies ranged from research involving IDIs of low-income single mothers to a quantitative study of cohabiting couples recruited via the Internet. The interview guide (Fig 2) covered the following domains: 1) day-to-day life and neighborhood context; 2) employment and career goals; 3) attitudes pertaining to relationships, marriage, etc.; 4) history and evaluation of current (or most recent) relationship; 5) childbearing; and 6) family formation. The interview ended with a final closed-ended “survey” question that asked the interviewee, “If you were voting today on whether same-sex marriage should be legal, would you vote in support of or against it?” The purpose of this final question was to explore the relationship between individual’s reported notions of “family values” from the interview data and their position on same-sex marriage at the societal level. This is particularly relevant given the subsequent legislation legalizing same-sex marriage in New York State, and is the subject of a separate report.

### Field-based project implementation

The SPAFF field staff included the principal investigator (PI), two doctoral student project coordinators, and 13 interviewers from various masters and doctoral social sciences and public health programs. The interviewers underwent a two-day training on in-depth interviewing and project management prior to entering the field. In order to further the interviewers’ skills with in-depth interviewing, each interviewer transcribed her first completed interview and submitted it to the PI for review. This allowed interviewers to further hone their data collection skills in a different manner (i.e. by listening to how they asked questions, probed [or not], and other aspects of their interaction with respondents). Conversely, the PI listened to the first several interviews conducted by each interviewer for monitoring/quality improvement. Constructive feedback was provided directly to interviewers and at team debriefing meetings, to be incorporated in the interviewing process going forward.

The recruitment and interview schedule consisted of three 4-hour shifts between 9:00 am and 8:00 pm seven days per week. The data collection period was 3.5 months, from 7 Feb 2011–17 May 2011. Interviewers worked in pairs in one or two of the five field sites. This allowed them to become familiar with their field site with a partner, collaborate on recruitment strategies, and explore additional recruitment venues. Each interviewer was expected to recruit potential project participants and conduct interviews. Recruitment involved approaching people at the selected venues, briefly explaining the project, and inviting them to complete the screener survey to determine their eligibility for the study. Those who completed the screener survey were given a Starbucks coffee gift card or a roundtrip NYC MetroCard (both worth approximately US$5) to thank them for their time. Following completion of the shift in the field, interviewers entered the relevant data online into a replica of the screener survey in SurveyMonkey. Upon approval from the project management team, the interviewer followed up to schedule the interview. Interviews were conducted either in interviewees’ homes, or in various public venues such as coffee shops or libraries. At the end of the hour-long IDI, study participants received US$45 cash.

### Project management

Given the large number of interviews being conducted, and the geographical dispersion of interviewers, it was imperative to maximize efficiency and continually monitor progress with the sampling and data collection activities. (Among other reasons, this is crucial to reducing threats to the validity of the data collected). A variety of pre-existing web-based tools were utilized in an integrated way to implement the sampling approach, enhance recruitment and facilitate management of field interviewers (Table 1). While each of the applications noted in Table 1 may be familiar as discrete tools for collaboration and project management, their combined use allowed real-time tracking of participants recruited from different neighborhoods, communication between 13 interviewers and project staff, exchange of information (e.g. recruitment strategies, study materials), and data entry, management and storage. Changes in web-based technologies have occurred since the time this study was in the field and continue to change; however, this provides a model for integration and use of currently available tools in the service of complex field data collection efforts. This is particularly useful since customized tools for these purposes can be costly, require broad-based buy-in, and may involve an extensive initial learning curve.

**Table 1:** Integrated use of web-based tools for field management and data collection.

Tool	Purpose	Benefits
Google Earth, Street View, My Maps	• Identify potential recruitment sites prior to field site visits • Create specialized site maps site • Included name/location of recruitment venues	• Interactive–all interviewers able to add venue suggestions • Virtual walk-throughs of selected neighborhoods
Google Group	• Primary mode of communication between project staff and interviewers	• Share recruitment strategies, post questions, address challenges, access archived experiences
Google Calendar	• Keep track of interviewers’ schedules	• Keep abreast of changing interviewers’ schedules • Support interviewer partnering in the field • Know when interview being done
Text Messages	• Safety measure: interviewers sent text message to coordinator when entering/leaving the field/interview	• Real-time means of communication
Google Docs	• Participant spreadsheet from screening through interview (e.g., ID#, location recruited, date screened, interview scheduled/completed)	• Real-time information from field • Track participants in need of follow-up
SurveyMonkey	• Centralize screening/eligibility data from interviewers • Track demographic distribution of screened participants	• Facilitate data entry • Data exports into SPSS • Can adjust recruitment follow-up based on sample distribution
Dropbox	• Share web-based storage for field materials (n.b. human subject guidelines at the time that this study was in the field permitted use of a cloud-based application which is no longer as secure as is required; other cloud-based storage sites with appropriate levels of data security should be utilized.)	• Share field materials (e.g., field manual, interview guides) • Interviewers can access additional documents (e.g., surveys, consent forms)
Skype	• Conduct (some) interviews to address scheduling conflicts; home-based concerns	• May recruit individuals who might decline in-person interview
Transcription	• Each interviewer transcribed their first interview	• Individual assessment of interviewing skill for improvement • PI mentoring of interviewers • Can adjust interview guide based on initial interviews

### Recruitment sites

**Fig 3:**
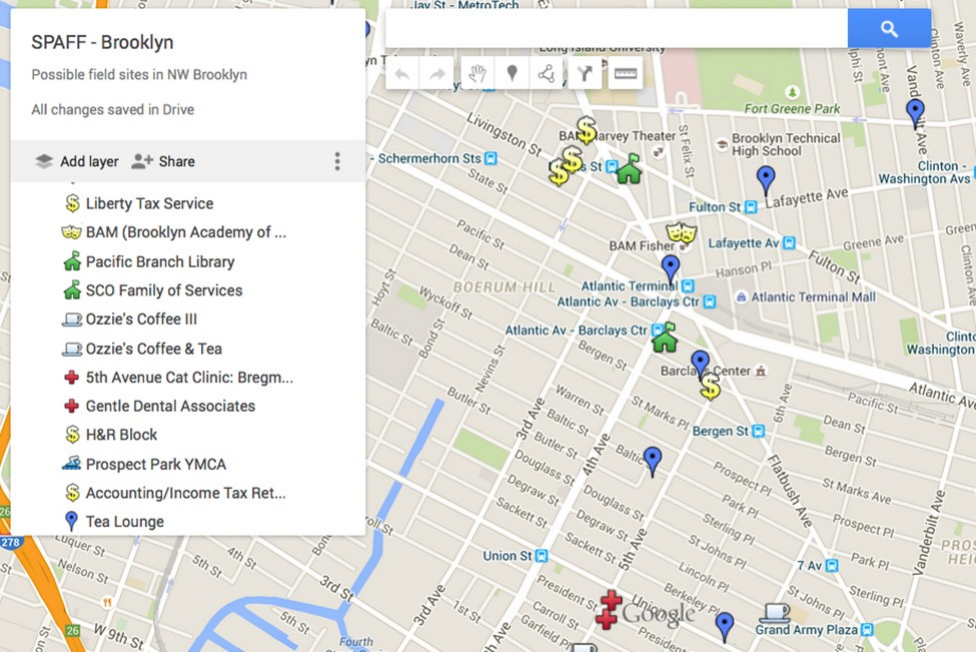
Sample custom Google map for a specific recruitment site in Brooklyn, NY.

We used Google Earth to “virtually look” at the potential recruitment sites that were initially identified through development of the sampling frame with the NYC and NJ demographic data described above. First, with the “Street View” function in Google Maps, we conducted virtual walk-throughs of the selected neighborhoods to identify well-trafficked commercial and residential areas that were likely to have multiple options for recruitment venues (e.g., public libraries, cafes, community centers). Using the “My Maps” function in Google Maps, we then created customized project maps of each recruitment site, including the names and locations of possible recruitment venues (Fig 3). These maps were shared virtually with the interviewers, who used these venue suggestions as starting-off points, but often found and shared other venues where recruitment was successful.

### Field staff communication

The interviewers were able to keep in touch with each other and the project staff through a Google Group. This Group was used to share suggestions for recruitment and interviewing, as well as to ask questions of the project staff that could easily be distributed to all of the interviewers at once. Since interviewers’ schedules had to be flexible to accommodate interviews at the convenience of study participants, they also entered their hours into a shared Google Calendar. The shared calendar allowed project management staff to track shifting schedules and also provided an easy way for interviewers to partner up in the field. Interviewers sent text messages to one of the project coordinators when entering and leaving the field as well as when beginning and ending interviews.

### Data collection and tracking

**Fig 4:**
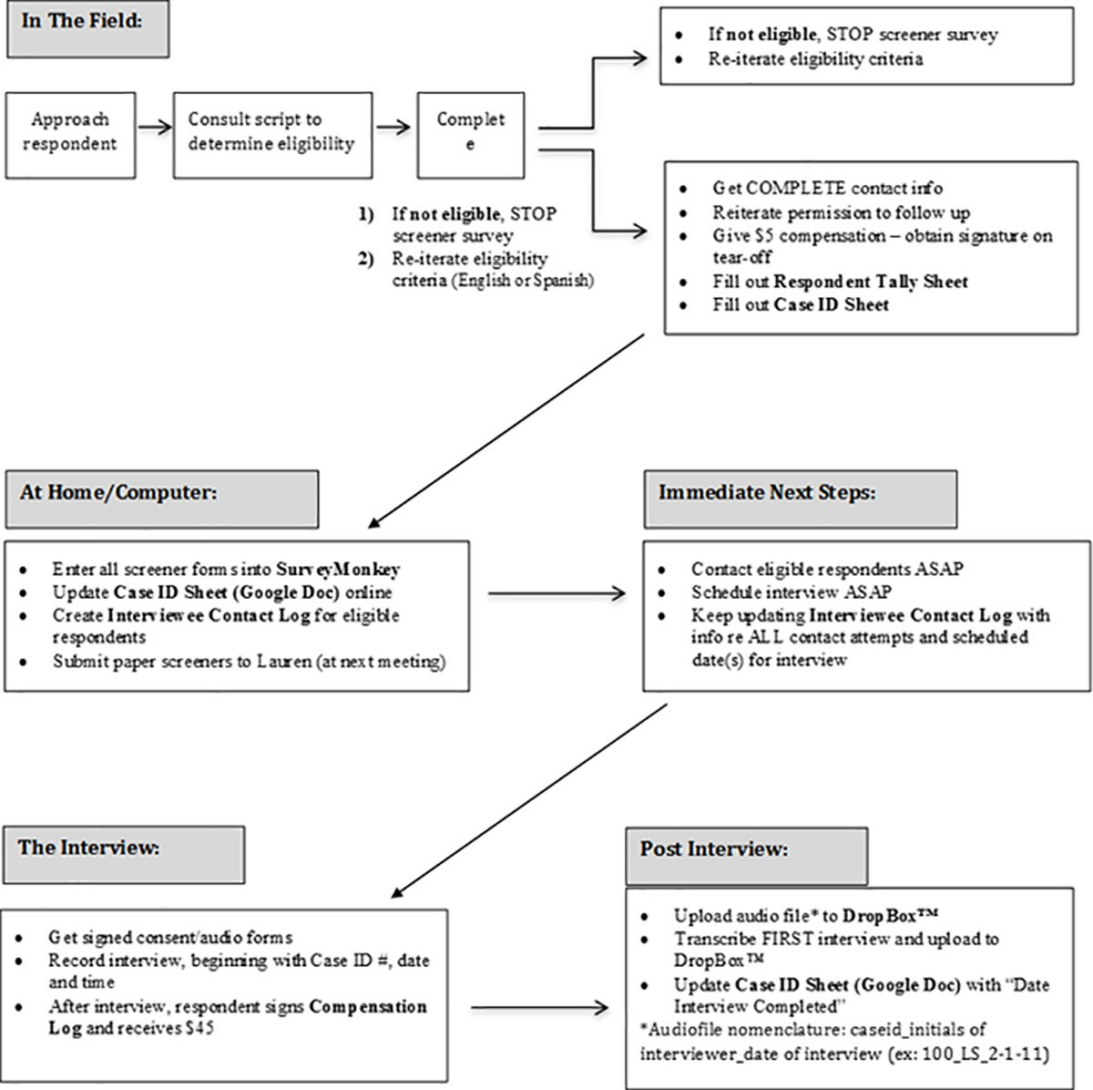
Flow chart to guide interviewers with data collection and field management.

Project staff used web-based tools such as Google Docs, Survey Monkey, and Dropbox to facilitate transfer of information from the field in as close to ‘real time’ as possible. Most importantly, screener data that interviewers entered into SurveyMonkey were exported to PASW Statistics 18 (IBM SPSS) by project staff. In this way, they were able to track demographic data as it was being collected in the field and make adjustments to recruitment parameters as needed (e.g., directing interviewers to recruit more men if the majority of those screened to date were women). Interviewers also entered newly recruited cases into a shared Google Docs spreadsheet. The spreadsheet tracked date of recruitment, scheduled date of interview, and date of interview completion, among other field-specific details. The data were uploaded with project-assigned ID numbers in place of personal identifiers in accordance with maintenance of the confidentiality of human subjects’ information. Using the shared spreadsheet, project coordinators could track field activity and interviewers were able to track their own progress. For example, the coordinators were able to follow up with interviewers regarding screened individuals for whom no date had been entered in the “Interview Scheduled” field, to assist with completion of the data collection process. A Dropbox (“cloud”) folder was created to virtually share project materials with interviewers and permit upload of audio files immediately upon completion of the interviews. These files were immediately relocated and stored in a more secure environment prior to transcription. We recognize that since the conduct of this project institutional guidelines have been put in place regarding requirements for electronic data storage. Despite use of a commercial cloud-based environment for this project, project-assigned ID numbers and participant-chosen pseudonyms were used in audio recordings in lieu of personal identifying information, providing protection regarding the identity of the sources of this confidential data. Using the Dropbox folder allowed interviewers to print additional forms (e.g. screener survey, consent forms, interview guide) from their respective locations as needed. Finally, two interviews were conducted using Skype videoconferencing for study participants who were unable to meet with the interviewer in a convenient and/or private location. A flow chart (Fig 4) was created to illustrate the many steps in the data collection process. This was included in the field manual to guide interviewers and minimize the likelihood of costly errors, potentially involving lost time, additional resources and valuable data.

### Analysis

The nature of this study is such that the analyses are being conducted in different yet complementary ways. The data collected via the IDIs pertain to the substantive research questions of this study and are qualitative in nature. Inductive thematic analysis using grounded theory methodology is being employed to identify key constructs pertaining to family-formation in the context of individuals’ social position [35–37]. These content-specific analyses are ongoing and will be reported separately in their complete form. Analysis of the quantitative sample description data pertains to the sampling methodology focus of this paper. Because the sampling approach employed to recruit a large, diverse community-based sample for IDIs [38–40] was informed by population characteristics in select parts of NYC and northern NJ, a key element of the analytic strategy sought to determine if the sample recruited met the goal of demographic and geographic diversity similar to the target boroughs in the greater NY Metropolitan area.

Data from the screener were downloaded from SurveyMonkey directly into PASW Statistics 18. The data were cleaned (i.e., electronic data inconsistencies reconciled by referring to the original field-based paper copies) and several variables were recoded (e.g., aggregation of relationship statuses such as combining separated and divorced). Descriptive analyses (frequencies, means) were run on all variables for the total number screened (n = 261), the sample that was interviewed (n = 200 participants) and those who were *not* interviewed (n = 61 non-participants). Bivariate analyses were conducted to identify potential differences between study participants and non-participants to identify potential participation bias.

## Results

The results of several different quantitative analyses of the sample are presented below. First, we provide recruitment data and a demographic description of the total sample interviewed. Next is the analysis of the study sample vis-à-vis the larger population from which it was drawn. Finally, we present the results of an analysis comparing study participants with those screened but who did not participate to detect potential participation bias. This is followed by a summary of the specific qualitative thematic analyses that are currently underway.

### Sample recruited and demographic characteristics

A total of 261 people were screened, of which four were ineligible. The interviews were conducted in a rolling fashion until 200 (76%) IDIs were completed. The recruitment and data collection period was 3½ months, which translates to approximately two interviews per day. The majority of the interviews were completed in public venues (63.5%) or in the interviewee’s home (35.5%). Two interviews were completed via Skype web-based video. The average length of interviews was 52 minutes. The sample was fairly evenly distributed by sex, age, neighborhoods, income, and race/ethnicity (Table 2). On average, participants were 27.6 years old (range: 18–35), with an almost equal distribution between women (52%) and men (48%). With respect to race/ethnicity, 36% identified as African-American/Black, 27% White, and 31% Hispanic. Almost 29% lived in Brooklyn, followed by the Bronx (20%), Manhattan (18%), Queens (17%) and northern New Jersey (16%). The majority reported a household income of US$20–39,999 (51%), with the other half approximately evenly divided between lower income (≤US19,999) and higher income (≥US$60,000). The educational distribution of the sample was quite diverse ranging from no high school diploma to a graduate degree, with the most common categories being some college and a Bachelor’s degree (both at approximately one third of the sample). (Data on educational attainment are based on ongoing analysis of interview data.) Participants selected the kind of relationship they are currently in from a list of six different statuses. The majority identified as being single (40%), followed by married (20%), living together (18%), in a committed (not married/living together) relationship (16%), divorced/separated (4%), and in an open relationship (2%). Approximately two thirds (64%) currently have no children; among those who did, 58% had one child, 23% had two, and 10% had three. Thus, overall, we achieved our objective of recruiting a diverse sample in terms of relationship/family composition, socioeconomic status, and other demographic characteristics.

**Table 2:** Sample description.

	Total Screened	Interviewed	Not Interviewed	p-value
	(n = 261)	(n = 200)	(n = 61)	
Variable	% (n)	% (n)	% (n)	
**Age (mean years)**	27.37 (SD = 4.63)	27.57 (SD = 4.55)	26.70 (SD = 4.85)	ns
	Range: 18–35	Range: 18–35	Range: 19–35	
	Median: 27	Median: 28	Median: 26	
	Mode: 26	Mode: 30	Mode: 25	
**Sex**				ns
Female	50.2 (131)	52.0 (104)	44.3 (27)	
Male	49.8 (130)	48.0 (96)	55.7 (34)	
**Race/Ethnicity**				.005
African-American/Black	31.4 (82)	35.5 (71)	18.0 (11)	
White	24.9 (65)	27.0 (54)	18.0 (11)	
Hispanic	34.5 (90)	30.5 (61)	47.5 (29)	
Asian/Pacific Islander	6.9 (18)	5.0 (10)	13.1 (8)	
Other/More than 1	2.3 (6)	2.0 (4)	3.3 (2)	
Borough*/City				ns
Brooklyn	25.7 (67)	28.5 (57)	16.4 (10)	
Bronx	21.1 (55)	20.0 (40)	24.6 (15)	
Manhattan	18.4 (48)	18.0 (36)	19.7 (12)	
Queens	19.2 (50)	17.5 (35)	24.6 (15)	
Jersey City	15.7 (41)	16.0 (32)	14.8 (9)	
**Annual Household Income**				.028
< = $19,999	27.7 (72)	23.6 (47)	41.0 (25)	
$20,000–59,999	48.5 (126)	50.8 (101)	41.0 (25)	
> = $60,000	23.8 (62)	25.6 (51)	18.0 (11)	
**Have Children**				ns
Yes	36.8 (96)	36.0 (72)	39.3 (24)	
No	63.2 (165)	64.0 (128)	60.7 (37)	
**Parity** (mean, SD)	.59 (0.98)	.55 (0.93)	.72 (1.11)	ns
**Current Relationship Status**				ns
Single	42.5 (111)	40.5 (81)	49.2 (30)	
Married	21.1 (55)	20.0 (40)	24.6 (15)	
Divorced/Separated	3.8 (10)	4.0 (8)	3.3 (2)	
Living together	16.9 (44)	18.0 (36)	13.1 (8)	
In a committed relationship	13.8 (36)	15.5 (31)	8.2 (5)	
In an open relationship	1.9 (5)	2.0 (4)	1.6 (1)	

ns = not significant; significant p values are indicative of a difference between those interviewed and not interviewed by the variable (i.e., race/ethnicity and income) but post-hoc analyses were not conducted to identify within which specific categories the differences exist

*Staten Island was not included in the sampling frame.

### Match between study sample and larger sampling frame

The main goal of the sampling approach applied was to recruit study participants from specific neighborhoods, from each of the four boroughs included, with demographic characteristics similar to that of the larger NY metropolitan area (neighborhoods described above). Although our non-random selection of boroughs and study participants prohibits generalizing findings to residents of NYC in these boroughs, we find it useful to investigate differences between our purposively drawn sample and datasets known to be representative of NYC neighborhoods. Our analyses and findings do not suggest that any non-significant differences between each sample is due to overlapping representativeness but rather that our quantitatively informed, methodological approach to purposively sampling respondents indeed produced aggregate demographic patterns similar to residents of non-randomly selected boroughs. In order to determine the extent to which we achieved that goal, we conducted a sub-group analysis of the racial/ethnic and income characteristics of the study sample by borough (Table 3) and then compared them to the population statistics (CHS data for the NYC boroughs and ACS data for Jersey City). For the income data, we compared the lowest income category in the study (≤US$19,999) with the percent in poverty from the population datasets. For almost every racial/ethnic and income category, the percent of the sample within most neighborhoods/boroughs of recruitment was very close (most within 6 percentage points) to the percent from the larger population. For example, the Hispanic sample in the study closely aligned with the larger NYC/NJ Hispanic population in the following way: 17.5% vs. 20% in Brooklyn; 25% vs. 27% in Manhattan; 25.7% vs. 25% in Queens; 60% vs. 48% in the Bronx; and 28.1% vs. 27.3% in Jersey City. Similarly, the percent in the lowest income category in the study closely mirrored the percent of NYC/NJ population at or below poverty: 21.1% vs. 25% in Brooklyn; 35% vs. 31% in the Bronx; 25% vs. 20% in Manhattan; 22.9% vs. 15% in Queens; and 12.5% vs. 15% in Jersey City.

**Table 3:** Key sampling variables by recruitment site compared to the NYC/NJ distribution.

Demographics	Brooklyn	Bronx	Manhattan	Queens	Jersey City
	% (n)	% (n)	% (n)	% (n)	% (n)
	(n = 57)	(n = 40)	(n = 36)	(n = 35)	(n = 32)
**Race/Ethnicity**					
AA/Black					
SPAFF sample	42.1 (24)	37.5 (15)	36.1 (13)	34.3 (12)	21.9 (7)
NYC-CHS/ACS*	*34*	*31*	*15*	*19*	*27*.*9*
White					
SPAFF sample	36.8 (21)	0 (0)	27.8 (10)	28.6 (10)	40.6 (13)
*NYC-CH/ACS*	*35*	*15*	*46*	*33*	*34*.*6*
Hispanic					
SPAFF sample	17.5 (10)	60.0 (24)	25 (9)	25.7 (9)	28.1 (9)
*NYC-CHS/ACS*	*20*	*48*	*27*	*25*	*27*.*3*
Asian/PI					
SPAFF sample	1.8 (1)	0 (0)	8.3 (3)	11.4 (4)	6.3 (2)
*NYC-CHS/ACS*	*7*	*3*	*9*	*17*	*20*.*1*
Other/>1					
SPAFF sample	1.8 (1)	2.5 (1)	2.8 (1)	0 (0)	3.1 (1)
*NYC-CHS/ACS*	4	3	3	6	—
**Annual Household Income**					
< = $19,999	21.4 (12)	35.0 (14)	25.0 (9)	22.9 (8)	12.5 (4)
% below poverty*	*25*	*31*	*20*	*15*	*15*
$20,000–59,999	57.1 (32)	50 (20)	47.2 (17)	45.7 (16)	50.0 (16)
> = $60,000	21.4 (12)	15.0 (6)	27.8 (10)	31.4 (11)	37.5 (12)

^*^Percentage from the *2006 Community Health Survey* for the respective NYC boroughs, and from the *2006–2008 American Communities Survey* for the Jersey City (NJ) data.

### Study participants interviewed vs. those not interviewed

We compared the demographic characteristics of the individuals who were interviewed (n = 200) with those who were screened but not interviewed (n = 61) to determine if there were significant differences between those who agreed to be interviewed and those who did not (i.e. participation bias) by carrying out a t-test to compare the mean age in the two groups and chi-square tests for all other categorical variables (Table 2). Overall, the study participants were very similar demographically to the individuals we did not interview. The groups differed on two variables—race/ethnicity and income. Specifically, relatively more African Americans and Whites, and relatively fewer Hispanics and Asians, agreed to be interviewed compared to the number screened. With regard to income, we interviewed significantly more middle- and higher-income individuals. That the sample contains relatively more White and affluent participants is reflective of the purposive sampling employed, given that the focus of much previous research has been on the poor and racial/ethnic minorities. Thus, with a broader racial/ethnic and income distribution in the sample, we can conduct thematic analyses both across and within groups. Specifically, there is an adequate amount of non-minority and higher income individuals to be analyzed, which heretofore have tended to be treated as the ‘referent’ group in quantitative studies examining factors related to family formation.

## Discussion

The complementary goals of this research project were to recruit a community-based sample for a large, IDI study that reflected key demographic characteristics of the target population. In order to do so effectively, we found that combined use of a host of web-based tools–from demarcating neighborhoods and identifying recruitment sites, to managing the field staff and collecting data–was integral to our success with recruiting a large, diverse sample for qualitative data collection.

### A quantitatively-informed purposive sampling approach for a large-scale IDI study

The purposive sampling strategy informed by key demographic characteristics of the larger populations (NYC, excluding Staten Island, and a site in northern NJ, respectively) enabled us to recruit individuals with a similar range of characteristics (e.g. racial/ethnic and economic backgrounds) from each neighborhood/recruitment site. In addition, the sampling strategy has enabled us to conduct sub-group analyses (reported elsewhere) by several factors relevant to family formation (e.g., relationship and parenting statuses) not typically possible from the same data of a qualitative study.

The SPAFF study also demonstrated that it is possible to conduct a large-scale, community-based qualitative study within a very short time frame. It took 3 ½ months to complete 200 IDIs, which is quite remarkable for a study requiring interviewers to approach strangers in public spaces (at the height of the winter season), invite them to participate in a social science study on personal relationships, and arrange for a separate hour-long interview oftentimes at their place of residence. We attribute this accomplishment to several factors. Foremost, we developed a thorough process for screening potential interviewers and an extensive training program. Once in the field, they were equipped with a detailed field manual that assisted with all of the elements of their work, including recruitment of study participants, conducting the interview, data management, and communication with the project management staff.

### Study implementation: Integral role of integrated use of web-based tools

The constellation of web-based tools that were incorporated into the study (from custom Google maps of recruitment neighborhoods, to real-time access to and review of the database of screened individuals, to upload of audio files for quality-control review by the PI) allowed for efficient processes, such that the time from individual participant recruitment to completion of the interview was very short (average of 10.7 days). There are several advantages to this, probably the most important being our ability to reduce the threat to validity associated with the occurrence of intervening events (referred to as “history” [41]), not within our control that could influence data collected from participants prior and subsequent to the event. We are not aware of any events that occurred during the 3 ½-month data collection period that would have been likely to influence individuals’ attitudes and/or behaviors pertaining to family formation.

Another important factor in conducting the field work was that the majority (11 of 13) of the interviewers remained on the project through completion of the data collection phase. In addition to the initial, comprehensive training in in-depth interviewing and field management, we provided interviewers with ongoing guidance to improve data collection. Some mechanisms included providing individualized feedback after reviewing interview audio files, regularly posting detailed instructions to the entire field staff on how to handle emerging issues via the Google Group site, and in-person “de-briefings” every few weeks for group members, which fostered shared problem-solving, camaraderie and continued excitement about the project. This resulted in experienced and efficient recruitment and data collection, as opposed to delays and poor data quality issues often associated with the typically high turnover rates among interviewers [42,43]. Thus, the high quality and consistent data collected from this skilled group increases confidence in the reliability of the data collected and ultimate validity of the findings.

## Conclusion

The SPAFF study utilized a research design involving both ‘quantitatively-informed’ purposive sampling and qualitative data collection, supported by combined use of various web- and field-based tools to address research questions pertaining to family-formation decision making. To best understand the dramatic changes in the configuration of relationships and childbearing patterns over the past half century [5,14,15], it was clear that much more extensive, qualitative data were needed. This sentiment was strongly articulated at a working group of family, reproductive and sexual health researchers and practitioners convened by the NIH National Institute of Child Health and Human Development [44]. To some researchers, large-scale qualitative data collection may be considered generally unnecessary and possibly antithetical to the paradigm of qualitative research. Yet, specific circumstances have been described in which larger sample sizes are valid and even necessary to address the research question(s) at hand [45]. Several of those conditions pertain to this study, including heterogeneity of the population; number of key selection criteria (including “nesting” of criteria, also referred to as stratification); groups of special interest that require intensive study; and, resources available. We felt that these conditions applied to the SPAFF study; thus, to best explore factors related to family-formation decision-making there was a compelling reason to undertake such a large-scale qualitative effort.

There is an extensive body of literature (particularly quantitative) on trends in family formation in the US, much focusing specifically on women (predominantly poor and low-income), and on racial/ethnic minorities [16,46–51]. Absent from many of these studies is substantive attention to men, whites and those with relatively higher incomes (except for use as statistical reference groups) [52]. Moreover, there is relatively less research on individual attitudinal and contextual factors underpinning these trends. Toward that end, the SPAFF study was designed to collect rich, in-depth data from a large sample of young heterosexual adults comprising diversity in gender, income, racial/ethnic identity, relationship and parental status. In this way, qualitative analyses of factors influencing family formation allows us to consider similarities and differences across these diverse groups within the same dataset. This offers the potential to contribute new knowledge in two ways. First, SPAFF data may help us better understand the factors underpinning population-level demographic changes. Second, the substantial portion of the SPAFF sample that is white, male, and more affluent attends to an area where there is currently a dearth of meaningful data and understanding of family formation behaviors.

To this end, we have conducted several stratified analyses of the interview data examining whether emergent themes vary by measures of social position, race/ethnicity and/or gender. In examining perceived ideal circumstances for childbearing across groups of different social position, we analyzed a subsample of individuals who became pregnant either before or during their perceived ‘ideal’ childbearing circumstances (n = 59) [53]. And given the demographic trend in delaying childbearing, another analysis has examined young adults’ attitudes regarding the ‘ideal’ age to have a first child and their understanding of the association of ‘advanced maternal age’ with fertility [54]. Other analyses of this large sample are underway. One seeks to understand how contemporary US parenting partnerships are formed and whether differences exist by gender, race/ethnicity and/or social position. Another analysis is exploring the effect of the financial burden of education on family-formation decisions among the subsample of young adult males (n = 96).

Because the validity, or trustworthiness, of study results is contingent on the quality and completeness of the dataset, the data collection methods employed must be carried out with the highest level of rigor. Our integrated use of web-based tools to support field management, data collection, and communication was instrumental in our ability to amass such a unique, large-scale dataset [55]. Consistent with the integrated use of technology in this study, we have utilized an academic social network/blog environment to support a collaborative data analysis approach inclusive of a web-based analytic software.

Our generation of a rich, qualitative dataset of 200 individuals, while not representative, reflects a distribution of demographic characteristics that aligns generally well with those of the larger populations from which they were recruited. This speaks to the success of the quantitatively-informed, purposive sampling approach we employed. This technique went beyond common purposive sampling approaches for qualitative data collection that typically do not consider the underlying distribution of key population characteristics. It is our hope that, depending on the specific research question, other qualitative researchers may find this sampling technique an improvement over convenience or purposive sampling methods alone (i.e., uninformed by examination of the population or other geographic or organizational units to achieve similar distributional patterns on key characteristics). Furthermore, we expect that the qualitative analyses of these data will similarly contribute new knowledge to the field of family demography and provide a deeper understanding of the ‘why’ and ‘how’ of family-formation decision-making among young adults of different socioeconomic position.
